# Friction and Stiffness Dependent Dynamics of Accumulation Landslides with Delayed Failure

**DOI:** 10.3390/e25071109

**Published:** 2023-07-24

**Authors:** Srđan Kostić, Kristina Todorović, Žarko Lazarević, Dragan Prekrat

**Affiliations:** 1 Geology Department, Jaroslav Černi Water Institute, Jaroslava Černog 80, 11226 Belgrade, Serbia; 2 Faculty of Mining, University of Banja Luka, Aleja kozarskog odreda 1, 79101 Prijedor, Bosnia and Herzegovina; 3 Faculty of Technical Sciences, University of Novi Sad, Trg Dositeja Obradovića 6, 21102 Novi Sad, Serbia; 4 Faculty of Pharmacy, University of Belgrade, Vojvode Stepe 450, 11221 Belgrade, Serbia; kristina.todorovic@pharmacy.bg.ac.rs (K.T.); dragan.prekrat@pharmacy.bg.ac.rs (D.P.); 5 China Road and Bridge Corporation Serbia, Užička 58-A, 11040 Belgrade, Serbia; lazarevic.zarko@gmail.com

**Keywords:** landslide monitoring, nonlinear time series analysis, determinism, bifurcation, stiffness, time delay, friction, homogeneity and heterogeneity

## Abstract

We propose a new model for landslide dynamics under the assumption of a delay failure mechanism. Delay failure is simulated as a delayed interaction between adjacent blocks, which mimics the relationship between the accumulation and feeder part of the accumulation slope. The conducted research consisted of three phases. Firstly, the real observed movements of the landslide were examined to exclude the existence or the statistically significant presence of background noise. Secondly, we propose a new mechanical model of an accumulation landslide dynamics, with introduced delay failure, and with variable friction law. Results obtained indicate the onset of a transition from an equilibrium state to an oscillatory regime if delayed failure is assumed for different cases of slope stiffness and state of homogeneity/heterogeneity of the slope. At the end, we examine the influence of different frictional properties (along the sliding surface) on the conditions for the onset of instability. Results obtained indicate that the increase of friction parameters leads to stabilization of sliding for homogeneous geological environment. Moreover, increase of certain friction parameters leads to the occurrence of irregular aperiodic behavior, which could be ascribed to the regime of fast irregular sliding along the slope.

## 1. Introduction

Landslides belong to a group of very frequent geological hazards that are in daily interaction with engineering activity—most commonly in three ways: (1) new construction is designed in an area which is endangered by landslides or is susceptible to the occurrence of landslides; (2) landslides threaten the existing structures; (3) landslides are activated due to engineering activities. All three possible cases are encountered daily, which makes the issue of modeling and predicting landslide activity one of the most important and, at the same time, most complex tasks in engineering and the corresponding scientific community. This problem of landslide activity could be solved by applying adequate prevention and/or remediation measures. However, in some cases, such measures do not provide enough safety from future occurrences of instability, or their application is not economically justified. Given this, the mutual action of monitoring and modeling could provide a satisfactory solution in the form of a reliable model, which could then be used to gain a deeper understanding of the mechanism of landslide triggering and dynamics or to predict landslide activation, size, and displacement range.

In general, landslide triggering is possible by act of natural or man-made factors, or because of their mutual interaction. The most significant natural factors that lead to landslide triggering are precipitation (rainfall, snow/snowmelt, and consequently floods) and earthquakes. Some of the largest landslides were triggered by earthquakes [1]. One should note that even small magnitude earthquakes may liquify (or partially liquify) the soil and lead to the occurrence or reactivation of landslides. In the case of accumulation landslides, as in the present paper, the main factor that induces the occurrence of landslides is the sudden reservoir discharge, when formed hydraulic gradient leads to the landslide activation. Such examples of accumulation landslide are frequent in engineering practice [2,3].

Landslide monitoring techniques during the last few decades developed significantly, and today usually combine the terrestrial methods (Light Detection And Ranging-LiDAR, inclinometers, piezometers, and geodetic benches) with air-borne (drones, aerial photogrammetry) and satellite interferometric synthetic-aperture radar ((In)SAR) approaches [4]. Based on the results of monitoring, one could apply different techniques in order to establish a certain pattern in the recorded time series, which will enable the derivation of a reliable prediction model. These methods usually include machine learning techniques [5,6], artificial neural networks [7], or approach based on Geographic Information System (GIS) [8]. Although most of these models provide satisfying prediction accuracy, one should note that such models are commonly site-based and cannot be used for the estimation of landslide activity at the location other than the one used for model derivation. Another group of models describes the general dynamics of landslides, and they serve for making deeper insight into the mechanism and dynamics of landslide activity. One of the first such models was presented in the work of Davis [9], who proposed an idealized model for certain classes of debris flows observed to exhibit sudden displacements. Results of his research indicated an approximate stability criterion for sudden movements of accumulation slides as a function of slope geometry, soil shear strength, and the ratio of masses of accumulation and feeder slides. In particular, Davis [9] analyzed a two-block model on an inclined slope, where the interaction between the blocks is idealized by a spring and dashpot.

In the present paper, we start from a model by Davis [9] and further expand it by assuming the delayed interaction among the accumulation (lower) and feeder (upper) part of the slope. Such delayed interaction was also observed by Davis [9] as a period between the onset of movement of feeder and accumulation part of the slope. Apparently, change in the water level of accumulation induces instability in the upper part of the slope which starts to slide, while the lower part of the slope remains in the equilibrium state. After some time, the load from the upper slide and oscillating groundwater level cause movement of the lower part of the slope. Apart from this possible delayed interaction between the upper and lower part of the accumulation slope, the assumed delayed interaction between the blocks could also be ascribed to the effect of delayed failure, as a result of time dependent weakening of clay soils due to loss of cohesion. For instance, according to Vaughan and Chandler [10], some short-time slope failures occurred during excavation while others occurred after a delay of about 50 days. Moreover, Skempton [11] noted delays in failure between 3 and 35 years for London clay slopes ranging in inclination from 1½:1 to 2¾:1 and in height from 6 to 17 m, and the delays in long-term failures to range from 46 to 65 years in slopes ranging from 3:1 to 2:31 and in height from 6 to 12.2 m.

Our model, inspired by the work of Davis [9] included the assumption made by Morales [12] regarding the nature of the friction law between the sliding blocks and the surface of the slope, which simulates the friction behavior along the sliding surface. The goal of the research presented in this paper is multifold:

- We want to show that the use of deterministic models for the analysis of landslide dynamics is justified, i.e., that the stochastic component does not have a significant impact on the landslide dynamics. This is achieved through analysis of the real observed landslide displacement by invoking the series of methods from nonlinear time series analysis.
- We propose a new model of landslide dynamics, where delayed failure is modeled by introduction of time delay in the position of two neighboring blocks, which mimic dynamics of the feeder and accumulation part of the slope (landslide). The use of time delay is additionally justified by previously determined delay in the dynamics of the accumulation and feeder part of the slope.
- Another goal is to examine the effect of different friction properties along the sliding surface on the landslide dynamics. This is achieved by assuming the same and different friction laws for the neighboring blocks. At the end, we want to determine the effect of the stiffness—susceptibility to occurrence of deformation—by analyzing the sole effect of spring stiffness and its mutual effect with time delay and friction.

One could say that in the present research, we are dealing with the landslide entropy, since Wang et al. [13] defined the entropy of a landslide as the extent of different landslide conditioning factors for landslide development, which is basically the main focus of the present research.

In order to achieve the aforementioned goals, we conducted the investigation in three phases. In phase 1, we analyzed the real-observed measurements of the landslide displacement to confirm that the sliding process could be modeled as a deterministic process and to exclude the existence or the statistically significant presence of background noise. For this, we use original recordings made at a single location of landslide for the 10-year period 2011–2020. In phase 2, once the deterministic nature of the landslide movement has been determined, we propose a new dynamical deterministic model for landslide dynamics, justified by the results of analysis in the first phase, that includes delayed failure and different friction laws. In this way, aside from the effect of the delayed failure, we also include the analysis of the influence of homogeneity/heterogeneity of the material that composes the slope. In this phase, we also analyze the effect of stiffness, which we consider as analog to the shear modulus of the slope. In phase 3, the effect of different frictional parameters, coupled with the introduced delay, is also examined.

## 2. Analysis of the Real Observed Data

**Figure 1:**
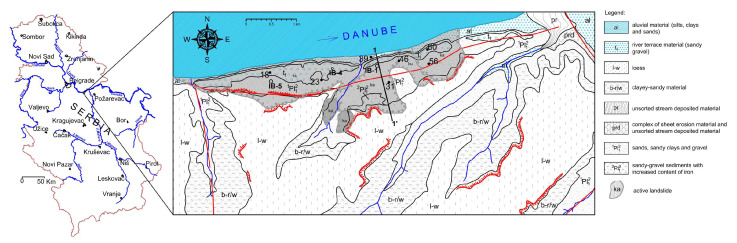
Location of the landslide, with engineering-geological map and distribution of the monitoring equipment. Red lines denote positions of faults, spiky lines denote the loess cliffs, points 18–60 denote the position of geodetic benches, point IB-1, IB-4, and IB-5 stand for the position of inclinometers.

We analyze the time series of the recorded data using geodetic benches (superficial movements) and inclinometers (movements along the depth) at the location of the landslide “Plavinac” in Smederevo (Figure 1). This landslide has been the subject of continuous monitoring since 2011, which also included conduction of the investigation of boreholes and terrain geophysical measurements. It should be emphasized that landslide activity at this location has been known for over 50 years, due to the combined effect of the erosion of the Danube River, groundwater level oscillation, and precipitation, including the impact of waste waters. The most significant influence comes from the groundwater level oscillations, since previous investigation indicated the existence of three aquifer horizons within the area of investigation: (1) the first aquifer with free level is formed within the superficial parts of terrain, in Quaternary deposits, (2) the second aquifer occurs in overlying sands with free level, and (3) the third aquifer is formed in underlying sands, and this aquifer is under pressure (artesian).

**Figure 2:**
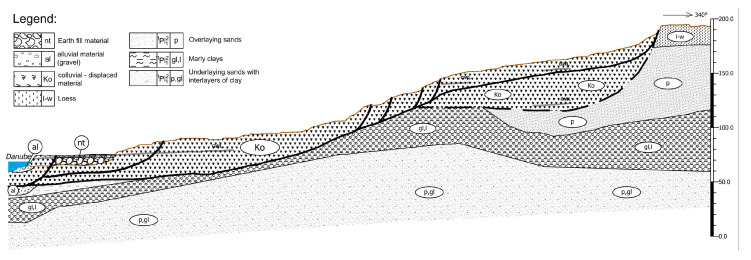
Typical engineering-geological cross-section 1-1’ in direction of landslide movement, as shown in Figure 1, according to data in [16].

Regarding the engineering-geological composition of the landslide, as one can see from Figure 2, colluvial material (Ko) is activated mainly in loess (l-w) and Pliocene sands (_1_PL_1_^2^—p), with the underlying layer of impermeable clayey-marley material (_1_PL_1_^2^—gl,l). The estimated depth of the sliding surface, depending on the terrain morphology, is in the range of 10–40 m. According to Varnes [14], this landslide belongs to the group of multiple translational earth slides, while according to Savarensky [15], it belongs to the group of insequent landslides, whose sliding surface cuts through different soil types.

**Figure 3:**
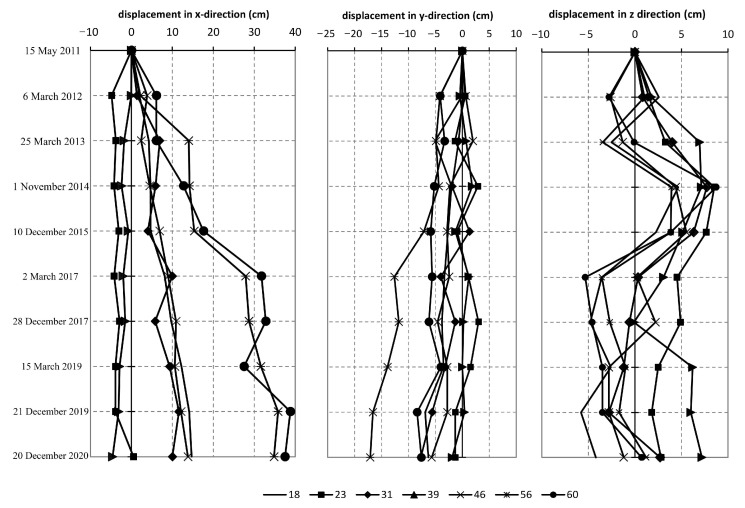
Time series of the superficial displacements recorded by geodetic benches in the period 2011–2020, at the location of landslide “Plavinac” in Smederevo [15].

**Figure 4:**
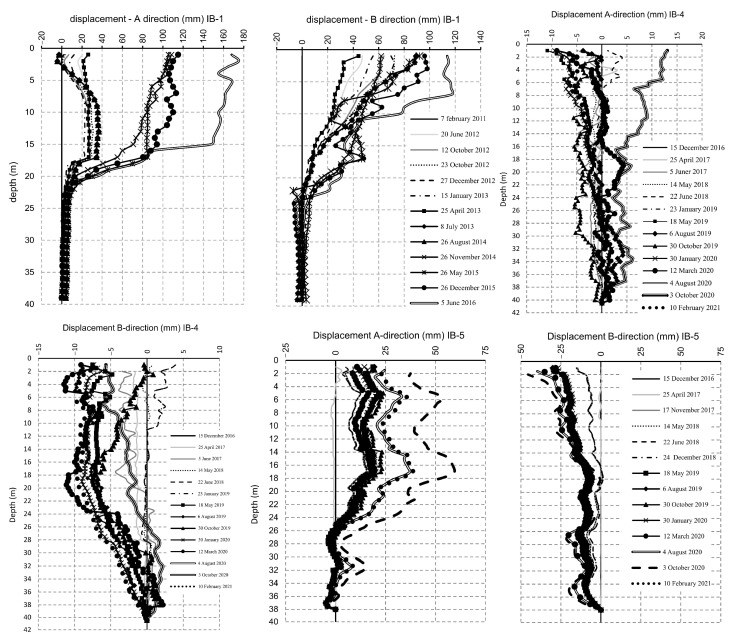
Recorded time series of the displacements along the depth, inclinometers IB-1, IB-4, and IB-5 [15].

In the present paper, we analyze the superficial movements recorded by geodetic benches (Figure 3) and displacements along the depth using inclinometers (Figure 4). The X-direction (increment +dX) denotes the displacements towards the north, while the Y-direction (increment +dY) denotes the displacements towards the east. The increment +dZ (Z direction) denotes the earth’s uprising, while -dZ stands for the settlement. As for the inclinometers, the A direction is oriented down the slope in the general direction of the landslide movement, while the B direction is perpendicular to the A direction.

The analysis of the recorded landslide displacements is conducted by invoking the methods of nonlinear time series analysis, which is a common example of an application of information-theoretic concept in engineering and complex systems. The final goal of the nonlinear time series analysis is to establish the value of the determinism factor *κ*, which is, according to Kaplan and Glass [17], a measure of the level of determinism/stochasticity of the observed system. The determinism factor is calculated using the determinism test, based on the assumption that if a time series originates from a deterministic process, it can be described by a set of first-order ordinary differential equations, whose vector field consists solely of vectors that have unit length. In other words, if the system is deterministic, the average length of all directional vectors will be 1, while for a completely random system, it will be 0.

In order to be able to determine this factor, we firstly need to embed the observed scalar series into the appropriate phase space according to Takens [18], which is done by calculating the values of optimum embedding dimension *m* and delay *τ* for all the recorded time series. The former is done by invoking the box-assisted method proposed by Schreiber [19], while the latter is done by applying the average mutual information method [20].

**Figure 5:**
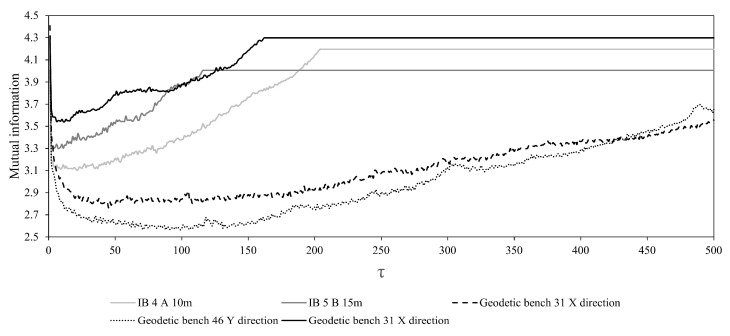
Results of mutual information method for some of the recorded time series. Qualitatively similar results are obtained for the rest of the examined series.

Results of the conducted time series analysis indicated the following. As one can see from Figure 5 and Table 1 and Table 2, determinism coefficient *κ* is in the range 0.46–0.95, with the average value of 0.75 (geodetic benches), and for the observed movements along the depth (inclinometers) *κ* is in the range 0.52–0.98, with the average value of 0.73 indicating high level of determinism in the observed time series.

It should be emphasized that one could consider the time series being analyzed in the present paper (shown in Figure 3 and Figure 4) as short ones, which is a challenging task and could eventually lead to biased results. However, we consider our results valid and non-biased for two main reasons. Firstly, a 10-year period of landslide monitoring could not be considered short, especially in the case when one has multidimensional data, as in this case. In particular, we analyzed landslide displacements by investigating three directions of displacements of seven spatially distributed superficial points, and movements along four different depths in two directions at three inclinometer locations. In total, we have a lot of different data at our disposal, and we consider such data to be sufficient for capturing the global trend of displacement, i.e., the goal is to establish whether the landslide movement is dominantly deterministic or stochastic. Secondly, the methods for the analysis of such data are adjusted to the size of time series and have previously been successfully used to analyze relatively short time series. For instance, Ma et al. [21] successfully used nonlinear time series methods to detect causality from nonlinear dynamics with short-term time series. Additionally, in practical situations, the measured time series are always limited rather than sufficiently long and sometimes are even rather short [22]. Moreover, in some cases, although long-term data can be measured, only short parts can correctly reflect the general dynamics of the system under study.

## 3. Description of the Proposed Mechanical Models

Once the significant stochastic impact on the landslide dynamics is excluded, concerning the obtained values of determinism coefficient *κ*, one could further approach studying the dynamics of landslide as a deterministic process. In the present paper, we start from the landslide model proposed by Davis [9]:

(1) $$m_{1}{\dot{V}}_{1}=W_{1}sin\beta_{1}-S_{1}-F\;m_{2}{\dot{V}}_{2}=W_{2}sin\beta_{2}-S_{2}+F\;\dot{F}=k\left(V_{1}-V_{2}\right)+c\left({\dot{V}}_{1}-{\dot{V}}_{2}\right)$$

where *W* is the block weight, *g* is the acceleration of gravity, *S* represents the sliding resistance on failure surface, *F* denotes the combined elastic and viscous forces, *k* is the spring constant, *c* is dash-pot constant, *V*_i_ represents the velocity of the i-th block, and *β_i_* is the slope angle.

Inspired by model (1), we propose the model for landslide dynamics in the following general form:

(2) $$\frac{dU_{n}}{dt}=V_{n}\;m\frac{dV_{n}}{dt}=k\left(U_{n+1}-2U_{n}+U_{n-1}\right)-F\left(V_{0}+V_{n}\right)+G$$

where *G* is the tangential component of the gravity force, and *F*(*V*) is a velocity-dependent friction force. A steady state of (2) exists when the block achieves a constant velocity motion *dU/dt = V*_0_, and then *F*(*V*_0_) = *G*, so Equation (2) represents a dynamical system moving at velocity *V*_0_. Model (2) actually represents an infinite chain of identical blocks linearly coupled through Hookean springs of stiffness k that slips at the constant velocity *V_0_* over an inclined surface.

For two coupled blocks, model (2) becomes:

(3) $$\frac{dU_{1}}{dt}=V_{1}\;m_{1}\frac{dV_{1}}{dt}=k\left(U_{2}-U_{1}\right)-F\left(V_{0}+V_{1}\right)+F\left(V_{0}\right)\;\frac{dU_{2}}{dt}=V_{2}\;m_{2}\frac{dV_{2}}{dt}=k\left(U_{1}-U_{2}\right)-F\left(V_{0}+V_{2}\right)+F\left(V_{0}\right)$$

Model (3) with the included delayed failure becomes:

(4) $$\frac{dU_{1}\left(t\right)}{dt}=V_{1}\left(t\right)\;\frac{dV_{1}\left(t\right)}{dt}=\frac{1}{m}\left[k\left(U_{2}\left(t-\tau \right)-U_{1}\left(t\right)\right)-F\left(V_{0}+V_{1}\left(t\right)\right)+F\left(V_{0}\right)\right]\;\frac{dU_{2}\left(t\right)}{dt}=V_{2}(t)\;\frac{dV_{2}\left(t\right)}{dt}=\left[k\left(U_{1}\left(t-\tau \right)-U_{2}\left(t\right)\right)-F\left(V_{0}+V_{2}\left(t\right)\right)+F\left(V_{0}\right)\right]$$

This model has already been discussed in the paper by Kostić et al. [23]. In the present paper, according to Morales [9], we assume that the sliding resistance along the failure surface has three different forms in two different geological conditions:

(1) Homogeneous geological conditions:

Case 1: both accumulation and feeder slope exhibit the Coulomb-like friction force along the surface of failure:

(5) $$\frac{dU_{1}\left(t\right)}{dt}=V_{1}\left(t\right)\;\frac{dV_{1}\left(t\right)}{dt}=\frac{1}{m}[k\left(U_{2}\left(t-\tau \right)-U_{1}\left(t\right)\right)-\left[1-\alpha +\sqrt{N\left(V_{0}+V_{1}\right)}\right]\frac{\left(V_{0}+V_{1}\right)}{\sqrt{\varepsilon +{\left(V_{0}+V_{1}\right)}^{2}}}\;+\left[1-\alpha +\sqrt{N\left(V_{0}\right)}\right]\frac{V_{0}}{\sqrt{\varepsilon +V_{0}^{2}}}]\;\frac{dU_{2}\left(t\right)}{dt}=V_{2}(t)\;\frac{dV_{2}\left(t\right)}{dt}=\frac{1}{m}[k\left(U_{1}\left(t-\tau \right)-U_{2}\left(t\right)\right)-\left[1-\alpha +\sqrt{N\left(V_{0}+V_{2}\right)}\right]\frac{\left(V_{0}+V_{2}\right)}{\sqrt{\varepsilon +{\left(V_{0}+V_{2}\right)}^{2}}}\;+\left[1-\alpha +\sqrt{N\left(V_{0}\right)}\right]\frac{V_{0}}{\sqrt{\varepsilon +V_{0}^{2}}}]$$

where $N\left(V\right)=\varepsilon +4max{\left(\left|V\right|-p,0\right)}^{2}+\alpha^{2}max{\left(p-\left|V\right|,0\right)}^{2}$ and *p*, *ε*, and *α* are dimensionless friction parameters. Parameter *p* is the location of the local minimum, i.e., the transition point from the velocity weakening to velocity strengthening regime. Parameter *ε* is a friction parameter which has a very small value, since friction function describes a regularized generalized Coulomb law as *ε*→0 [12]. Parameter *p* is chosen to have a very small value, *p* = 1, and its effect on the dynamics of the observed system is not examined.

Case 2: both accumulation and feeder slope exhibit the cubic friction force along the surface of failure:

(6) $$\frac{dU_{1}\left(t\right)}{dt}=V_{1}\left(t\right)\;\frac{dV_{1}\left(t\right)}{dt}=\frac{1}{m}[k\left(U_{2}\left(t-\tau \right)-U_{1}\left(t\right)\right)-\left[a{\left(V_{0}+V_{1}\right)}^{3}-b{\left(V_{0}+V_{1}\right)}^{2}+c\left(V_{0}+V_{1}\right)\right]\;+a{\left(V_{0}\right)}^{3}-b{\left(V_{0}\right)}^{2}+cV_{0}]\;\frac{dU_{2}\left(t\right)}{dt}=V_{2}(t)\;\frac{dV_{2}\left(t\right)}{dt}=\frac{1}{m}[k\left(U_{1}\left(t-\tau \right)-U_{2}\left(t\right)\right)-\left[a{\left(V_{0}+V_{2}\right)}^{3}-b{\left(V_{0}+V_{2}\right)}^{2}+c\left(V_{0}+V_{2}\right)\right]\;+a{\left(V_{0}\right)}^{3}-b{\left(V_{0}\right)}^{2}+cV_{0}]$$

where *a*, *b*, and *c* are friction parameters.

(2) Heterogeneous geological conditions, where feeder slope exhibits the Coulomb-like friction force, while accumulation slope exhibits the cubic friction force:

(7) $$\frac{dU_{1}\left(t\right)}{dt}=V_{1}\left(t\right)\;\frac{dV_{1}\left(t\right)}{dt}=\frac{1}{m}\left[k\left(U_{2}\left(t-\tau \right)-U_{1}\left(t\right)\right)-\left[1-\alpha +\sqrt{N\left(V_{0}+V_{1}\right)}\right]\frac{\left(V_{0}+V_{1}\right)}{\sqrt{\varepsilon +{\left(V_{0}+V_{1}\right)}^{2}}}+\left[1-\alpha +\sqrt{N\left(V_{0}\right)}\right]\frac{V_{0}}{\sqrt{\varepsilon +V_{0}^{2}}}\right]\;\frac{dU_{2}\left(t\right)}{dt}=V_{2}(t)\;\frac{dV_{2}\left(t\right)}{dt}=\frac{1}{m}\left[k\left(U_{1}\left(t-\tau \right)-U_{2}\left(t\right)\right)-\left[a{\left(V_{0}+V_{2}\right)}^{3}-b{\left(V_{0}+V_{2}\right)}^{2}+c\left(V_{0}+V_{2}\right)\right]+a{\left(V_{0}\right)}^{3}-b{\left(V_{0}\right)}^{2}+cV_{0}\right]$$

By assuming different types of friction laws, we introduce the homogeneity or heterogeneity of the geological material into the calculation. In particular, the Coulomb-like friction law corresponds well to brittle materials—harder soil or weakly cemented rocks, such as marlsontes or claystones, which exhibit very small deformation before the failure (almost without any plastic deformation), followed by a long period of frictional healing. On the other hand, cubic friction force mimics the behavior of more ductile materials, such as clay, marls, or silts, which show significant plastic deformation before the failure point. Further combination of these two friction laws within a single model brings additional complexity and heterogeneity and describes the cases when failure surfaces occur through different geological materials (the so-called “insequent landslides”).

## 4. Results

In all examined cases, equilibrium state is considered as the state of steady small displacement with constant velocity, while the occurrence of the first Hopf bifurcation and transition to periodic or quasiperiodic oscillations is considered as a point of instability.

The analysis of dynamics of proposed models (5)–(7) is conducted numerically, for constant values of some of the control parameters, in order to determine the conditions for the instability to occur, under the effect of the introduced time delay τ and spring stiffness k, as well as under the impact of different frictional parameters. For this purpose, we use the Runge–Kutta 4th order numerical method. One should note that numerical solving of delay differential equations is commonly followed by the issue of numerical instability, in which case some other numerical methods are invoked, such as backward differentiation formula if the analyzed system is numerically stiff. Nevertheless, in the present case, numerical instability was not encountered.

### 4.1. Influence of Delay and Spring Stiffness

**Figure 6:**
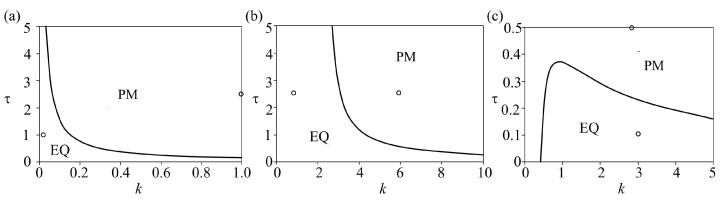
Bifurcation diagrams k-τ for models (4)–(6): (**a**) model (4)—both slides exhibit Coulomb-like friction force (*ε* = 10^−4^, *p* = 1, *α* = 0.2, *V*_0_ = 0.2; initial conditions: *U*_1_ = 0.001, *U*_2_ = 0.0001; *V*_1_ = *V*_2_ = 0.1), (**b**) model (5)—both slides exhibit cubic friction force (*a* = 3.2, *b* = 7.2, *c* = 4.8; initial conditions: *U*_1_ = 0.001, *U*_2_ = 0.0001; *V*_1_ *= V*_2_ = 0.1), (**c**) model (6)—feeder slope exhibits Coulomb-like friction force, accumulation slope exhibits cubic friction force. Parameter values and initial conditions are the same as in (**a**,**b**). EQ stands for equilibrium state (steady movements of low intensity), while PM denotes the regime of regular periodic oscillations.

**Figure 7:**
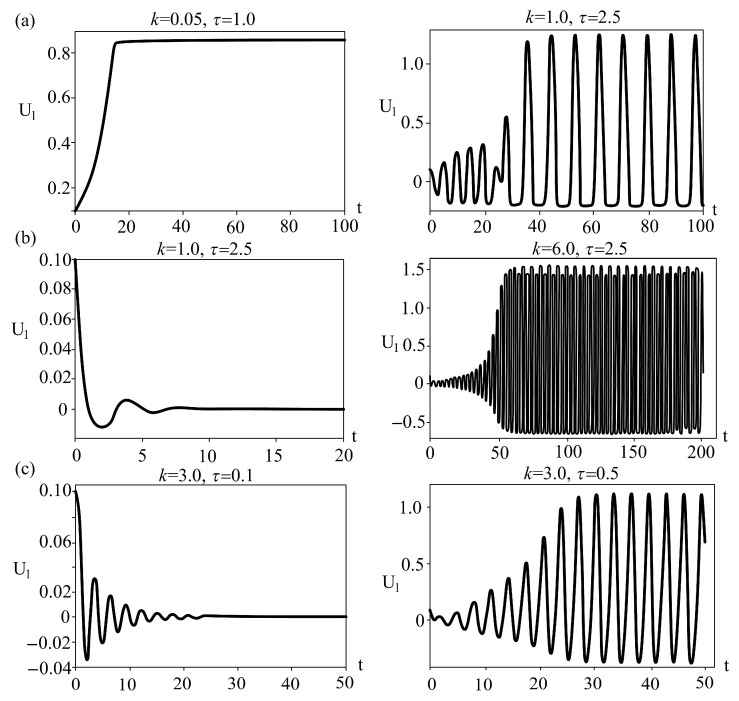
Characteristic time series for the (*k*,*τ*) points as marked in Figure 6: (**a**) refers to points in Figure 6a, (**b**) refers to points in Figure 6b, (**c**) refers to points in Figure 6c.

Results of the analysis of the effect of time delay and spring stiffness are shown in Figure 6, while the corresponding time series are given in Figure 7. The analysis was done for the constant values of control parameters: *a* = 3.2, *b* = 7.2, and *c* = 4.8 for the cubic friction force, which correspond to the local minimum of the function, where the local minimum is the transition point from the velocity-weakening (*b < V < a*) to the velocity-strengthening (*V > a*) regime, as proposed by Morales [8]. For Coulomb-like friction force, we assumed small value of parameter *ε, ε* = 10^−4^.

Results of the performed analysis indicated the occurrence of the Hopf bifurcation, indicating a transition from equilibrium state to periodic regular oscillations, for all three examined dynamical systems. From a viewpoint of geodynamics, the observed dynamics could be interpreted in the following way. As shown in Figure 6a, when a slope prone to sliding is made of homogeneous brittle material, introduction of time delay between the movement of the feeder and the accumulation part of the slope causes instability for almost all values of spring stiffness. The greater the stiffness, the lower the value of τ required to “provoke” the instability. Regarding the sliding process in situ, these results could be interpreted in the following way: (a) high spring stiffness values correspond to fresh unweathered or slightly weathered rock masses (marlstone/claystone), where almost any delay in the movement of the upper and lower parts of the slope would result in brittle failure and the occurrence of instability; (b) low spring stiffness values correspond to loosely bonded upper and lower parts of the slope, which could be the case when the entire slope or a portion of it was subjected to intense weathering and superficial alteration; in this case, delay in the movement between two parts of the slope is expected and it does not necessarily lead to the occurrence of instability (see EQ region for *k* < 0.1). The latter is a very common case in engineering practice, since marlstones and claystones are prone to weathering, with the possibility of forming up to a 20-m-thick weathering crust.

Figure 6b clearly shows that the effect of time delay is much lower when the slope is predominantly composed of plastic material (clays, silts, or marls). As one can see for *k* < 3, the delay between the motion of the upper and lower parts of the slope has absolutely no effect on the occurrence of instability. This could be explained by the ductility of the material, which can withstand large plastic deformation before the actual failure occurs. Higher values of spring stiffness correspond to the cases when the upper and lower parts of the slope are in a tighter connection, which is the case when the ductility of the material decreases and brittleness increases, making the transition to case 1 (Figure 7a). Certainly, cases involving more ductile materials (lower values of spring stiffness) are more common in engineering practice. 

As for Figure 6c, when the slope is composed of heterogeneous material, one can observe unusual dynamics. For extremely low values of spring stiffness, indicating the case when the upper and lower parts of the slope are composed of different materials (brittle/ductile), the system under study is in the unstable regime even without the time delay. This could indicate the case when the shear strength parameters of the material are so low that no equilibrium could be established between its own weight (under the act of gravity forces) and shear strength. These are the landslides whose movements are continuously being observed throughout the year, such as landslides Plavinac, Vinča, Ritopek, and similar in Serbia [16]. However, with the increase of spring stiffness, which indicates the decrease of the difference between the upper and lower parts of the slope, the system under study is in an equilibrium regime only for the low values of time delay. This means that a heterogeneous slope is more sensitive to the effect of delayed movement between the upper and lower parts of the slope, regardless of the spring stiffness.

If one looks at the effect of spring stiffness, it could be seen that when slope is composed of homogeneous material, the increase of *k* leads to the onset of instability. In the case of real sliding in situ, this means that the tighter connection between the upper and lower parts of the slope causes failure when the delay between the movement of different parts of the slope is considered. This is expected since the increased slope stiffness indicates the response of all parts of the slope at approximately the same time, while the delayed reaction triggers the increase of shear stress, which overcomes the shear strength along the potential sliding surface, leading to the occurrence of instability. In the case when heterogeneous material composes the slope, one can see the following two possible scenarios: for low values of delay, the increase in spring stiffness actually leads to stabilization of the landslide, which is expected since the tighter connection between the blocks indicates a unique response to destabilizing forces. On the other hand, for slightly higher values of delay, an increase in *k* does not have any impact on the dynamics of the slope.

### 4.2. Influence of Friction Parameters

**Figure 8:**
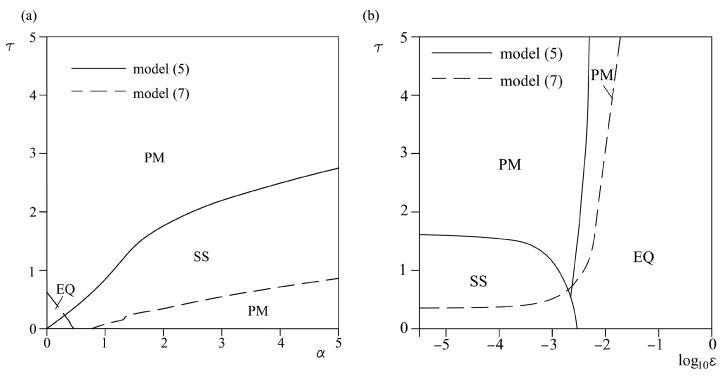
Bifurcation diagrams *τ-α* (**a**) and *τ-ε* (**b**) for models (5) and (7). While *τ*, *ε*, and *α* are varied, other parameters are being held constant: *ε* = 10^−4^, *p* = 1, *α* = 0.2, *V_0_* = 0.2, *a* = 4.8, *b* = −7.2, *c* = 3.2, initial conditions: *U*_1_ = 0.001, *U*_2_ = 0.0001; *V*_1_ *= V*_2_ *=* 0.1. EQ denotes the steady state (no motion), SS marks the steady sliding, and PM denotes the occurrence of periodic oscillations.

Results from the analysis of the effect of Coulomb friction parameters on the landslide dynamics, i.e., models (5) and (7), are shown in Figure 8. The friction parameter *α* has a different effect on a homogeneous model (5) than on a heterogeneous model (7). For homogeneous model (5), increase of *α* leads to stabilization of sliding, i.e., transition from oscillatory regime to stable sliding. On the other hand, for the heterogeneous model (7), increase of *α* leads to a destabilization of sliding, but only up to *τ* = 1. For higher values of *τ*, change of this friction parameter does not have any effect on the dynamics of the model (7). Regarding the effect of friction parameter *ε*, both for models (5) and (7), increase of these parameters leads to a transition from an oscillatory regime or stable sliding to an equilibrium state.

**Figure 9:**
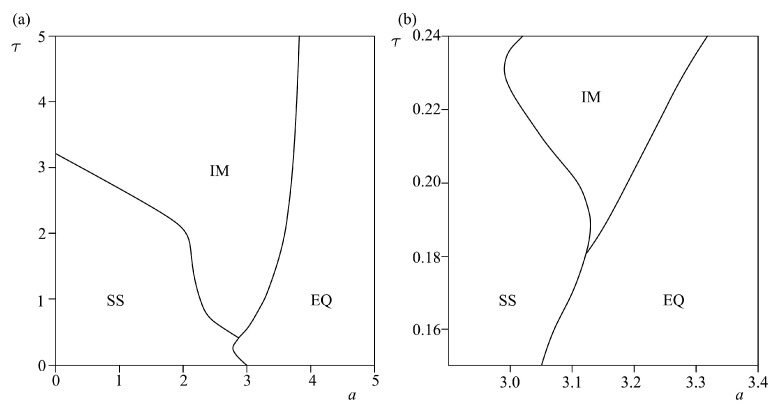
Bifurcation diagrams *τ-a* for models (6), (**a**) and (7), (**b**). While *τ* and *a* are varied, other parameters are being held constant: *ε* = 10^−4^, *p* = 1, *α* = 0.2, *V*_0_ = 0.2, *b* = −7.2, *c* = 3.2, initial conditions: *U*_1_ = 0.001, *U*_2_ = 0.0001; *V*_1_ *= V*_2_ = 0.1. EQ denotes the steady state (no motion), SS marks the steady sliding, PM denotes the occurrence of periodic oscillations, while IM stands for irregular oscillations.

**Figure 10:**
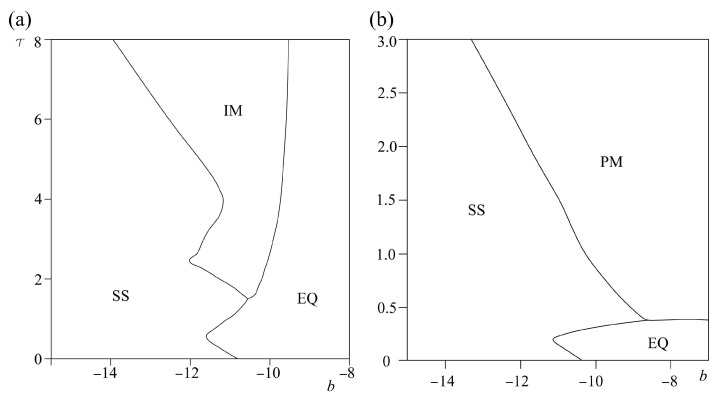
Bifurcation diagrams τ-b for models (6), (**a**) and (7), (**b**). While *τ* and *b* are varied, other parameters are being held constant: *ε* = 10^−4^, *p* = 1, *α* = 0.2, V_0_ = 0.2, *a* = 4.8, *c* = 3.2, initial conditions: *U*_1_ = 0.001, *U*_2_ = 0.0001; *V*_1_ *= V*_2_ = 0.1. EQ denotes the steady state (no motion), SS marks the steady sliding, PM denotes the occurrence of periodic oscillations, while IM stands for irregular oscillations.

**Figure 11:**
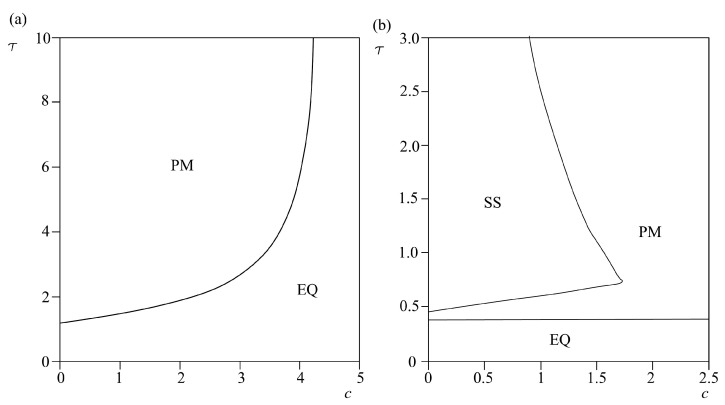
Bifurcation diagrams *τ-c* for models (6), (**a**) and (7), (**b**). While *τ* and *c* are varied, other parameters are being held constant: *ε* = 10^−4^, *p* = 1, *α* = 0.2, V_0_ = 0.2, *a* = 4.8, *b* = −7.2, initial conditions: U_1_ = 0.001, U_2_ = 0.0001; V_1_ = V_2_ = 0.1. EQ denotes the steady state (no motion), SS marks the steady sliding, and PM denotes the occurrence of periodic oscillations.

The effect of cubic friction parameters on homogeneous model (6) and heterogeneous model (7) is shown in Figure 9, Figure 10 and Figure 11. The following phenomena are observed:

- There is an occurrence of irregular aperiodic behavior, which could be treated as an example of real sliding along the slope. Such a dynamical regime, denoted as IM in Figure 9, occurs with the increase of parameter *a* both for homogeneous and heterogeneous models, and with the increase of parameter *b* for homogeneous model.

- Increase of all parameters *a*, *b*, *c* leads to the transition of model (6) and (7) to equilibrium state, except in the case when parameters *b* and *c* are increased in heterogeneous model (7), when the system under study transitions to a periodic dynamical regime. This is a similar effect to the effect of parameter *α* in model (7), indicating that an increase of friction in the case of complex friction behavior does not necessarily lead to the stabilization of sliding.
- It could be concluded that friction parameters *a* and *b* have qualitatively the same influence on the dynamics of the models (6) and (7) since the increase of these parameters leads to transition from stable sliding to irregular aperiodic motion to an equilibrium state.
- The friction parameters *b* and *c* qualitatively have the same influence on the dynamics of the heterogenous model (7), since the change of these parameters leads to the occurrence of stable sliding, periodic motion, or leads the observed systems to an equilibrium state.
- The increase of friction parameter *c* in model (6) leads to stabilization of the homogeneous model, i.e., brings the system from periodic motion to an equilibrium state.

## 5. Discussion

From the geodynamic point of view, the results of the performed analysis indicate the following. When a homogeneous slope prone to sliding is built on fresh unweathered rock masses, such as marlstones or claystones, then a delay in interaction between the feeder and accumulation slope is not expected. Nevertheless, if it occurs, then instability is triggered almost for any value of time delay, confirming the brittle behavior of the material, which is sensitive to any disruption in the response of the slope to the act of shear forces. If the slope is composed of weathered rock masses, then delay between the feeder and accumulation slope is expected, and it does not necessarily lead to the onset of instability, i.e., higher values of delay are required to trigger the instability, indicating more ductile behavior of the slope.

In cases when the homogeneous slope is composed of plastic material (clay, silts, or marls), the effect of delay between the feeder and accumulation slope is felt only in the cases of stiffer slope, indicating the property of the plastic material to exhibit large plastic deformation before the actual instability occurs. As the plasticity of the material decreases (the rise of consistency index), the slope becomes more sensitive to the delay between the feeder and the accumulation slope.

When heterogeneous material builds the slope, results of analysis show that heterogeneous material with very low stiffness (deformation modulus) is always in an unstable state. This state is expected when the slope is composed both of brittle and plastic-ductile material. With the increase of slope stiffness, the difference in mechanical behavior between the ductile and brittle parts of the slope decreases (both parts are either ductile or brittle), and the slope enters the equilibrium regime—slow steady movement. It should be emphasized that such behavior is observed only for very low values of time delay (below 0.4). For higher values of delay, the heterogeneous slope is always in an unstable state, regardless of the time delay and stiffness.

When it comes to the effect of slope stiffness, for the slope composed of homogeneous material, an increase in the slope stiffness leads to the onset of instability only if delayed interaction is assumed, due to an increase of shear stress acting along the potential siding surface. When heterogeneous material composes the slope, the increase in spring stiffness could lead to landslide stabilization for low values of time delay, while the dynamics of a landslide are independent of the slope stiffness for higher (and reasonable) values of time delay.

The effect of the change of friction parameters on the dynamics of models (5)–(7) revealed the following. Four typical dynamical regimes occur: equilibrium state (complete stabilization—no motion), stable sliding (stationary movement), periodic oscillatory regime, and irregular aperiodic behavior. Increase of all the examined friction parameters, i.e., *α* and *ε* for Coulomb friction and *a*, *b*, and *c* for cubic friction leads to stabilization of sliding in the case of homogenous models (5) and (6). This indicates that sliding in a homogeneous geological environment is strongly dependent on the friction along the potential sliding surface. Moreover, this further indicates that, in the case of old landslides whose movement was temporarily interrupted, they could be permanently stabilized if friction would increase with time, due to higher bonding of clay particles or effects of consolidation. On the other hand, when it comes to heterogeneous model (7), an increase of friction parameters, such as α in Coulomb friction, or *b* and *c* in cubic friction, could lead to final transition to an unstable oscillatory regime, which indicates that friction properties along the potential sliding surface do not play a crucial role in the stabilization of sliding in a heterogeneous geological environment. Apart from this, one needs to emphasize the occurrence of irregular aperiodic behavior for certain values of parameter *a* (both for homogeneous and heterogeneous models) and with the increase of parameter *b* for a homogeneous model.

If one compares these results with the results of our stochastic landslide model [24], it is clear that the deterministic model exhibits more reach dynamic behavior than the stochastic one, confirming the predominant deterministic nature of the landslide dynamics. Moreover, it is shown that complex landslide dynamics could be triggered and observed with a much simpler model compared to the work of Chau [25], who also studied the nonlinear dynamical model of landslide dynamics, but with two-state variable friction law, where friction is logarithmically dependent on the velocity. Moreover, irregular aperiodic motion which is captured in *τ-a* (Figure 9) and *τ-b* (Figure 10) bifurcation diagrams could indicate the onset of deterministic chaos, which was previously indicated as the inherent dynamical regime of unstable landslide dynamics [26].

## 6. Conclusions

In the present paper, we propose a new model for the dynamics of an accumulation landslide in the form of an interconnected two-block setup moving along the sliding surface. The interaction of these two blocks simulates the relationship between the feeder (upper) and accumulation (lower) parts of the slope. The main novelty of the presented research and its major originality are as follows:

- For the first time, results of the monitoring of landslide dynamics are examined by invoking the set of methods from nonlinear time series analysis, in order to confirm the predominant deterministic nature of the landslide motion.
- Results of our research confirmed that both sliding at the ground surface and along the depth of the sliding body could be considered as deterministic. This a practical confirmation that our deterministic approach is justified.
- For the first time, we suggest a spring-block model of the landslide dynamics, which includes:
- the effect of delayed failure, introduced as delayed interaction of two blocks,
- slope stiffness, as a measure of resistance to shear force and deformation, examined as variable spring stiffness, and
- the homogeneity/heterogeneity of the material that composes the slope, analyzed through different friction properties along the contact of blocks and the sliding surface.
- Results obtained indicate that the increase of friction parameters does not lead to the stabilization of sliding in a heterogeneous geological environment, indicating that friction properties along the potential sliding surface do not play a crucial role in stabilization of sliding in a heterogeneous geological environment.
- It is determined that the increase of certain friction parameters leads to the occurrence of irregular aperiodic behavior, which could be ascribed to the regime of fast irregular sliding along the slope. This irregular aperiodic regime could be treated as a real example of unstable sliding along the slope, and further characterization of this regime and conditions for its occurrence should be investigated.
- Regarding the results of the nonlinear time series analysis of the recorded landslide displacements, the main limitations could come from the relatively short time series, which could also be treated as scarce when observing only the recordings of superficial movements, where only one recording per year was available for the analysis. Concerning this, further analysis should include verification of the results obtained in the present study. This should be done by investigating the updated time series (from continuous monitoring) or by testing the longer time series from some other location within the same landslide, or at different landslides. In our opinion, results of these further studies will not change the main conclusion of the present study, which indicated the predominant deterministic nature of the landslide dynamics, but could provide more insight into the remaining stochastic term. Such studies could further enable the analysis of the effect of small-amplitude background noise or seismic impact on the landslide dynamics, which could lead to a transition between different dynamical regimes and eventual occurrence of irregular aperiodic behavior. Additionally, further research could be directed to the investigation of the occurrence and properties of transient behaviors and their significance for landslide dynamics.

Additionally, regarding the results of nonlinear timeseries analysis, one should note that two different obtained values of embedding dimension (2 and 3) for different values of embedding delay may indicate the need for the use of non-uniform embedding over uniform embedding [27], which should also be verified in further studies.

- From the viewpoint of nonlinear dynamics and the suggested model, accuracy of the suggested model should be observed as qualitative, meaning that we are only interested into the conditions for which proposed dynamical system of landslide movement goes from one dynamical regime to another. The performed analysis is dimensionless, and it is focused on the main mechanism behind the landslide dynamics, rather than providing exact quantitative values of the main factors that trigger the unstable landslide motion. One should note that the main limitations of the proposed model come from the fact that the proposed model does not reflect the spatial relations within the slope, i.e., blocks in the model are not connected with other blocks in the space. Such additional complexity in future studies could potentially lead to the occurrence of new dynamic features.

## Figures and Tables

**Table 1:** Results of nonlinear time series analysis for recordings made at geodetic benches, location of “Plavinac” landslide (Smederevo, Serbia).

Monitoring Location	Direction of Displacement	Embedding Delay τ	Embedding Dimension m	Determinism Coefficient κ
No. 18	Along x axis	2	2	0.72
	Along y axis	5	2	0.63
	Along z axis	4	2	0.94
No. 23	Along x axis	11	3	0.64
	Along y axis	6	2	0.67
	Along z axis	1	2	0.46
No. 31	Along x axis	7	2	0.70
	Along y axis	5	2	0.75
	Along z axis	2	2	0.90
No. 39	Along x axis	12	2	0.57
	Along y axis	8	3	0.69
	Along z axis	3	2	0.91
No. 46	Along x axis	8	2	0.76
	Along y axis	8	2	0.50
	Along z axis	1	2	0.88
No. 56	Along x axis	1	2	0.93
	Along y axis	5	2	0.77
	Along z axis	2	2	0.87
No. 60	Along x axis	4	2	0.86
	Along y axis	5	2	0.68
	Along z axis	5	2	0.95

**Table 2:** Results of nonlinear time series analysis for recordings made at inclinometers, location of “Plavinac” landslide (Smederevo, Serbia).

Monitoring Location	Direction of Displacement	Measurement Depth (m)	Embedding Delay τ	Embedding Dimension m	Determinism Coefficient κ
IB-1	A	5	8	2	0.65
		10	2	2	0.58
		15	5	2	0.54
		20	7	2	0.79
	B	5	10	2	0.84
		10	6	2	0.62
		15	10	2	0.66
		20	7	3	0.65
IB-4	A	5	3	2	0.71
		10	4	2	0.52
		15	1	3	0.72
		20	3	2	0.96
	B	5	3	3	0.81
		10	2	2	0.57
		15	3	2	0.69
		20	4	2	0.89
IB-5	A	5	3	2	0.84
		10	7	2	0.65
		15	5	2	0.67
		20	3	2	0.98
	B	5	2	2	0.77
		10	3	3	0.70
		15	1	2	0.75
		20	4	2	0.94

## Data Availability

The datasets generated during and/or analyzed during the current study are not publicly available due to privacy restrictions but are available from the corresponding author on reasonable request.

## References

[1] Ferrario M.F. (2019). Landslides triggered by multiple earthquakes: Insights from the 2018 Lombok (Indonesia) events. Nat. Hazards.

[2] Guo Z., Chen L., Yin K., Shrestha D.P., Zhang L. (2020). Quantitative risk assessment of slow-moving landslides from the viewpoint of decision-making: A case study of the Three Gorges Reservoir in China. Eng. Geol..

[3] Wang R., Wan J., Cheng R., Wang Y., Wang Z. (2023). Physical and Numerical Simulation of the Mechanism Underpinning Accumulation Layer Deformation, Instability, and Movement Caused by Changing Reservoir Water Levels. Water.

[4] Scaioni M. (2014). Modern technologies for landslide monitoring and prediction. Springer Nat. Hazards.

[5] Huang F., Huang J., Jiang S., Zhou C. (2017). Landslide displacement prediction based on multivariate chaotic model and extreme learning machine. Eng. Geol..

[6] Zhang T., Han L., Chen W., Shahabi H. (2018). Hybrid Integration Approach of Entropy with Logistic Regression and Support Vector Machine for Landslide Susceptibility Modeling. Entropy.

[7] Yang B., Yin K., Lacasse S., Liu Z. (2019). Time series analysis and long short-term memory neural network to predict landslide displacement. Landslides.

[8] Shahabi H., Hashim M. (2015). Landslide susceptibility mapping using GIS-based statistical models and Remote sensing data in tropical environment. Himan Shahabi & Mazlan Hashim. Sci. Rep..

[9] Davis R.O. (1992). Modelling stability and surging in accumulation slides. Eng. Geol..

[10] Vaughan P.R., Chandler H.J. (1974). Notes concerning informal discussion on ‘The Design of Cuttings in Overconsolidated Clay’ at the Institution of Civil Engineers, London.

[11] Skempton A.W. Slope stability of cuttings in brown London clay, Special Lectures. Proceedings of the 9th International Conference on Soil Mechanics and Foundation Engineering.

[12] Morales J.E.M., James G., Tonnelier A. (2017). Travelling waves in a spring-block chain sliding down a slope. Phys. Rev. E.

[13] Wang Q., Li W., Wu Y., Pei Y., Xie P. (2016). Application of statistical index and index of entropy methods to landslide susceptibility assessment in Gongliu (Xinjiang, China). Environ. Earth Sci..

[14] Varnes D.J., Schuster R.L., Krizek R.J. (1978). Slope movement types and processes. Special Report 176: Landslides: Analysis and Control.

[15] Janjić M. (1979). Engineering Geodynamics.

[16] Jaroslav Černi Water Institute (2021). Expert Opinion for the Purpose of Reissuing the Water Permit of HPP “Iron Gate 1”.

[17] Kaplan D., Glass L. (1992). Direct test for determinism in a time series. Phys. Rev. Lett..

[18] Takens F., Rand D.A., Young L.S. (1981). Detecting strange attractors in turbulence. Lecture Notes in Mathematics 898.

[19] Schreiber T. (1995). Efficient neighbor searching in nonlinear time series analysis. Int. J. Bif. Chaos.

[20] Fraser A., Swinney H. (1986). Independent coordinates for strange attractors from mutual information. Phys. Rev. A.

[21] Ma H., Aihara K., Chen L. (2014). Detecting Causality from Nonlinear Dynamics with Short-term Time Series. Sci. Rep..

[22] Balacco G., Alfio M.R., Parisi A., Panagopoulos A., Fidelibus M.D. (2022). Application of short time series analysis for the hydrodynamic characterization of a coastal karst aquifer: The Salento aquifer (Southern Italy). J. Hydroinf..

[23] Kostić S., Vasović N. (2022). Delay-Resilient Dynamics of a Landslide Mechanical Model. Nonlinear Dynamics and Applications: Proceedings of the ICNDA 2022.

[24] Kostić S., Vasović N., Todorović K., Prekrat D. (2023). Instability Induced by Random Background Noise in a Delay Model of Landslide Dynamics. Appl. Sci..

[25] Chau K.T. (1999). Onset of natural terrain landslides modeled by linear stability analysis of creeping slopes with a two-state variable friction law. Int. J. Numer. Anal. Methods.

[26] Huang Z., Law K.T., Liu H., Jiang T. (2009). The chaotic characteristics of landslide evolution: A case study of Xintan landslide. Environ. Geol..

[27] Ragulskis M., Lukoseviciute K. (2009). Non-uniform attractor embedding for time series forecasting by fuzzy inference systems. Neurocomputing.

